# Prevalence of Marijuana Use among University Students in Bolivia, Colombia, Ecuador, and Peru

**DOI:** 10.3390/ijerph120505233

**Published:** 2015-05-15

**Authors:** Marya Hynes, Maria Demarco, Juan Carlos Araneda, Francisco Cumsille

**Affiliations:** 1 Inter-American Observatory on Drugs, Inter-American Drug Abuse Control Commission (CICAD), Organization of American States (OAS), 1889 F Street NW, Washington, DC 20006, USA; E-Mails: mdemarco@oas.org (M.D.); fcumsille@oas.org (F.C.); 2 Global SMART Programme (Latin America), United Nations Office on Drugs and Crime, 1889 F Street NW, Washington, DC 20006, USA; E-Mail: jaraneda@oas.org

**Keywords:** marijuana, drug use, university students, Latin America

## Abstract

Young adults 18 to 25 years old show the highest prevalence of marijuana use in Latin America. This study analyzes the changes in prevalence of marijuana use among university students in the Andean Community (Bolivia, Colombia, Ecuador, and Peru) from two studies carried out in 2009 and in 2012. Data were collected through representative two-stage samples of universities and students in the Andean Community. An online survey was administered using a standardized questionnaire. Prevalence was calculated for lifetime, past year, and past month. Marijuana was the most widely used illicit substance consumed among university students, in 2009 and in 2012. Past month prevalence among university students in 2009 in Colombia was 5.27%, in Peru 1.00%, in Ecuador 1.68%, and in Bolivia 0.76%. Past month prevalence in 2012 in Colombia was 7.14%, in Ecuador 3.67%, in Peru 1.62%, and in Bolivia 1.45% in 2012. Among university students in the Andean Community, past month prevalence increased among both males and females between 2009 and 2012 in most countries. Marijuana continues to be the most commonly used illicit drug in Latin American countries. Increases in prevalence among young adults could have important implications for national drug policy.

## 1. Introduction

It is estimated that during 2012, between 125 million and 227 million people world-wide, between the ages of 15 and 64, used marijuana in their lifetime, corresponding to 2.7%–4.9% of the world population [1]. Furthermore, approximately 10% of first time users and 50% of daily users showing signs of dependence [2]. Marijuana abuse is common among adolescents and positively correlated with continued use during adulthood [3].

Marijuana is the most commonly used illicit drug in the American Hemisphere [4]. General population surveys carried out in the Andean Community showed that the past year prevalence of marijuana use in the population age 12–64 years was 2.3% in Colombia (2008), 0.7% in Peru (2006), 4.5% in Bolivia (2007), and 0.7% in Ecuador (2007) [4]. Furthermore, the highest prevalence of drug use in each country is found among young adults aged 18–25 years old. In studies on drug use in the general population, young adults aged 18–34 years old had past year prevalence of marijuana use as high as 11.5% in Colombia, 4.9% in Peru, and 17.2% in Bolivia [4].

There is a great deal of speculation in the current debate on the liberalization of drug use laws in the Americas regarding how changes in laws across countries may contribute to the changes in prevalence of marijuana use. For this reason, it is extremely important for countries to monitor drug use over time and understand with clarity both the time and the circumstances in each country relative to those changes. Most countries in Latin America do not have time series data on drug use trends [4]. Some countries that are able to show trend data over time have serious limitations regarding the frequency of their drug use surveys and the number of years of data available. Nevertheless, national studies from drug use surveys in different populations give the impression that there has been a gradual rise in marijuana prevalence in Latin America, coupled with a decrease in the perception of risk associated with marijuana use, and increase in the perception of availability, which have been highly influential in current drug policies in Latin America [4].

The purpose of this paper is to analyze patterns of marijuana use among male and female university students in the Andean Community (Bolivia, Colombia, Ecuador, and Peru), and report the changes in prevalence between 2009 and 2012. Given the recent changes in marijuana legislations in the US and public perception throughout the Hemisphere [4], we hypothesize that there will be an increase in the prevalence of marijuana use from 2009 to 2012 in all countries, both for males and females. This is the first study comparing nationally-representative samples of university students in all Andean countries. Our findings could be instrumental in the conceptualization of patterns of drug use among university students in the region.

## 2. Methods

### 2.1. Sampling

Data for this analysis were collected in two independent studies conducted in 2009 and 2012, using a standard methodology [5,6]. The base population was students in public and private universities in each Andean country (Bolivia, Colombia, Ecuador, and Peru), in cities with population of 300,000 or higher, with 60% or more urban population. Representative, two-stage, randomly selected samples were obtained for 21,857 students in 37 private and public universities in 2009, and 22,389 students in 45 universities in 2012. Stage one was the random selection of universities according to the above inclusion criteria. In stage two, a random sample of students at each university was taken, in order to ensure a representative sample of students within each university. Statistical weights and adjustments for complex sample designs were used to ensure generalizability of results to the target population.

In each study, an online survey was administered using a standardized questionnaire. The questionnaire includes a variety of questions on the patterns of use of licit and illicit drugs, such as alcohol, marijuana, cocaine, inhalants, and synthetic drugs. Socio-demographic variables are included, as well as indicators for perception of risk associated with drug use, and ease of access to drugs. The questionnaire incorporates scales to define criteria for signs of drug dependence, signs of drug abuse, and measures of anxiety and depression.

Participation in these surveys was voluntary, and informed consent was obtained from all students participating in the survey. Exclusion criteria included students who did not agree to the informed consent and those who did not fully complete the questionnaire. The software used for the questionnaire did not allow respondents to leave any questions unanswered, resulting in no missing values for any of the variables.

No identifiable information was collected from participants. Therefore, it was not possible to tell what proportion of the survey respondents were the same individuals from one year to the next. However, each student selected for the sample, was given a unique user name and password to submit their answers in the system, so participants could only participate and respond once.

### 2.2. Measures

Prevalence was measured through the questions: “Have you used marijuana in your lifetime?”, “Have you used marijuana in the past year?”, and “Have you used marijuana in the past month?” Prevalence variables were calculated dividing the number of people who used marijuana in a given time, over the total number of students surveyed.

A proxy for incidence is included in the questionnaire, “When was the first time you tried marijuana?” permitting an estimate of the proportion of new users during three time frames: past month, past year, and over a year. Incidence variables were defined as the proportion of students who used marijuana for the first time in the period chosen, among those who had never used marijuana until then.

# Of people who reported using marijuana for the first time during the previous 12 months =%

Total number of people at risk of initiating marijuana use

Marijuana use prevalence were calculated for subgroups of the study population. Significant differences between users in 2009 and 2012 were reported based on *p*-values of 0.01 when appropriate. Sampling weights were used to produce national estimates. All results were considered significant based on 95% confidence intervals. SAS version 9.3 and SPSS 20 were the statistical software used for all analyses.

## 3. Results

Marijuana was the most widely used illicit substance among university students, although prevalence varied across the Andean countries (Table 1). Changes in prevalence in the four countries were characterized by increases in lifetime, past year and past month prevalence in each country and across gender groups. Differences in prevalence between 2009 and 2012 were statistically significant for all countries, in the overall population and among males. Differences in prevalence for females were only significant in Colombia and Ecuador. In Bolivia, lifetime prevalence of marijuana use increased from 7.49% in 2009 to 11.97% in 2012, past year prevalence increased from 2.04% to 3.44%, and past month prevalence increased from 0.76% to 1.45%. In Colombia lifetime prevalence increased from 26.41% in 2009 to 31.16% in 2012, past year prevalence increased from 11.51% to 15.01% and past month prevalence increased from 5.27% to 7.14%. In Ecuador, lifetime prevalence increased from 11.41% in 2009 to 21.94% in 2012, past year prevalence increased from 4.43% to 9.00% and past month prevalence increased from 1.68% to 3.67%. In Peru, lifetime prevalence of marijuana use increased from 8.40% in 2009 to 11.58% in 2012, past year prevalence increased from 2.97% to 4.29%, and past month prevalence increased from 1.00% to 1.62%. Table 1 illustrates the increase in past month prevalence of marijuana use between 2009 and 2012, by country.

**Table 1:** Prevalence of marijuana use in Andean countries *.

		Bolivia		Colombia		Ecuador		Peru	
		2009	2012	2009	2012	2009	2012	2009	2012
**Lifetime**	Males	11.09	19.65	32.83	39.03	17.25	32.37	10.51	16.88
	Females	3.76	6.44	19.27	24.35	6.18	13.10	5.61	6.57
	**Total**	**7.49**	**11.97**	**26.41**	**31.16**	**11.41**	**21.94**	**8.40**	**11.58**
**Past year**	Males	2.66	5.06	14.55	19.86	6.88	12.64	3.36	6.02
	Females	1.40	2.27	8.15	10.81	2.24	5.93	2.46	2.65
	**Total**	**2.04**	**3.44**	**11.51**	**15.01**	**4.43**	**9.00**	**2.97**	**4.29**
**Past month**	Males	0.94	2.45	6.76	10.26	2.10	5.52	1.08	2.66
	Females	0.56	0.73	3.61	4.44	1.30	2.10	0.91	0.63
	**Total**	**0.76**	**1.45**	**5.27**	**7.14**	**1.68**	**3.67**	**1.00**	**1.62**

* Differences in prevalence between 2009 and 2012 were statistically significant (*p* < 0.01) for all countries, in the overall population and among males. Differences in prevalence for females were only significant in Colombia and Ecuador.

We observed differences in the patterns of use among males and females, with males showing higher prevalence in all countries (Table 1). The magnitude of this difference varied between countries, with larger gender gaps in Ecuador, Peru and Bolivia. Increases in prevalence between 2009 and 2012 were observed for both males and females in all countries except Peru. In Bolivia, past month prevalence almost doubled from 2009 to 2012, with an increase from 0.94% to 2.45% among males and from 0.56% to 0.73% among females. Colombia showed a more moderate increase from 6.76% to 10.26% among males and from 3.61% to 4.44% among females. Ecuador was also close to doubling its prevalence by gender in 3 years, with changes from 2.1% to 5.52% among males and from 1.3% to 2.1% among females. In contrast with these patterns, prevalence of past month use of marijuana increased among males in Peru from 1.08% to 2.66% but appears to have decreased from 0.91% to 0.63% among females.

While marijuana use increased for males and females in most countries, the gaps in marijuana use prevalence seem to be closing over time. Increase in marijuana prevalence among females could be related to a decrease in perception of risk, increase in perception of ease of access, or a raise in prevalence among younger cohorts. While perception of risk of marijuana use in Peru is similar for males and females, the decrease in prevalence could be partially explained by the lower perception of ease of access among females.

**Figure 1:**
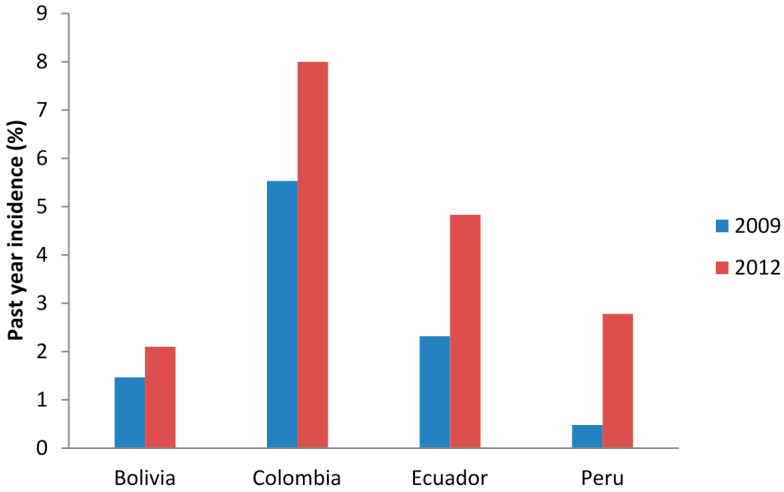
Past year incidence of marijuana use in Andean Countries, by country.

Incidence of marijuana use during the past 12 months rose in all four of the countries; however, the degree of change in incidence varied. In Figure 1 we can observe that the past year incidence of marijuana use in Bolivia was 1.47% in 2009 and 2.1% in 2012. Incidence increased in Colombia as well from 5.53% to 8.00%. Indeed, in 2012 almost half of past year users in Colombia used marijuana for the first time in the 12 months prior to the survey. Incidence of marijuana use nearly doubled in Ecuador from 2.32% to 4.83%, and increased fivefold in Peru from 0.48% to 2.78%.

## 4. Discussion

Drug use studies among university students focus on people 18−25 years old, which is the age group that has been demonstrated to have highest prevalence of drug use in the general population [4]. However, despite the prevalence rates observed among young adults aged 18−25, there is currently very little knowledge on their patterns of drug use and perceptions. The online application of the CICAD University Student Survey methodology allows for easy access to information and convenient monitoring over time [5]. Previous studies among university students using the CICAD methodology have prompted the participating universities to develop their own school-wide drug prevention policies [7].

Although prevalence in the four Andean countries is quite diverse, there are some common elements across countries that merit reflection. In all four countries, prevalence is higher for men than for women, however, marijuana use increased among both men and women between 2009 and 2012 in every country except for Peru. These data reflect a broader gender gap in drug prevalence in this age group than we see in countries such as the United States and Canada, where marijuana prevalence among males and females is very similar. Nevertheless, the rise in marijuana use among females in the university population of these countries should be recognized.

This study evidences the generalized increase in prevalence of marijuana use among university students in the Hemisphere and contributes to the theories of a recent increase in marijuana use in the last decade. While we cannot establish causal relationships, the timing of his increase in prevalence is paired with changes in public opinion and debates on marijuana legislations throughout the Americas. These changes might be coupled with changes in perception of risk and perception of ease of access that could have an effect on marijuana use among university students.

Differences in prevalence between countries and by gender seem to be partially explained by perception of risk and availability. In our study, prevalence of marijuana use was lower among sub-populations who perceived high risk of occasional marijuana use, and low ease of access. This pattern was applicable for differences by gender, with males having higher prevalence, higher perception of easy access, and lower perception of great risk, as well by country. No known factors have been associates with the decline in prevalence among females in Peru.

The incidence rates in this study indicate that in Colombia approximately half of the past-year users, used for the first time during the previous 12 months. Scientific literature on the health effects of marijuana use has demonstrated that marijuana acute intoxication has a negative effect on learning, memory, attention, concentration and abstract reasoning [8]. These impacts persist even when the individual is not presently using the substance. The cognitive processes involved in learning and impulse control are also risk factors for drug dependence [8]. This raises a significant issue for universities if their students are initiating use while attending.

The primary limitation of this analysis is that we only have only two years of data. In the absence multiple years of study or time series data, it is impossible to determine with any level of certainty whether the changes we observe represent a growing trend. However, given that other drug use surveys in Latin America have been showing systematic increases in marijuana use over time, these changes in prevalence should be taken seriously. In Peru, the one country with without an increase in marijuana use among women between 2009 and 2012, we are unable to say whether this will be meaningful over time given that we have only two data points to examine. Furthermore, given that we are using data from two independent studies conducted in 2009 and 2012, some of the differences reported from one year to the other might be due to sampling differences, cohort effects, or response rates. The four countries are planning a third round of surveys to be carried out in 2015−2016. This will provide better indicators on the trends in use over time.

## 5. Conclusions

The data from the surveys in the four Andean countries could be used for more in-depth analysis. Areas that merit further examination include the dynamics of drug use among females. The national databases also contain demographic information, and more detailed information on issues such as anxiety, stress and other possible risk factors for drug use, leaving open possibilities for more secondary analyses for this population.

The countries that participated in these studies can use them to develop more focused and relevant policies on drug use. The universities that participated each retained a copy of their own data-base. It is hoped that they will carry out further analysis of these data in order to respond effectively to their particular situation. Ultimately, either the governments of the four countries, or the universities themselves, or perhaps both will need to decide whether or not to develop policies and interventions to address drug use in their populations.
